# Detecting apple replant disease in the field – deciphering reasons for local growth depression

**DOI:** 10.1371/journal.pone.0345851

**Published:** 2026-04-21

**Authors:** Anne-Sophie Wachter, Kristin Hauschild, Benye Liu, Patrick Schnoor, Elke Bloem, Andreas Wrede, Rolf Hornig, Ludger Beerhues, Steffen Schlüter, Kornelia Smalla, Traud Winkelmann, Doris Vetterlein

**Affiliations:** 1 Helmholtz Centre for Environmental Research – UFZ, Halle Saale, Germany; 2 Julius Kühn Institute (JKI) - Federal Research Centre for Cultivated Plants, Institute for Epidemiology and Pathogen Diagnostics, Braunschweig, Germany; 3 Technische Universität Braunschweig, Braunschweig, Germany; 4 Chamber of Agriculture Schleswig-Holstein, LKSH, Ellerhoop, Germany; 5 Julius Kühn Institute (JKI) - Federal Research Centre for Cultivated Plants, Institute for Crop and Soil Science, Braunschweig, Germany; 6 LMS agricultural consultation, Rostock, Germany; 7 Leibniz University Hannover (LUH), Hannover, Germany; Amity University Mumbai, INDIA

## Abstract

Apple replant disease (ARD) arises from repeated apple (*Malus domestica*) planting in the same area, disrupting physiological and morphological plant functions. Recent studies demonstrated that ARD occurs locally with low mobility in soil. The patchy distribution of ARD makes field identification of its severity difficult. Moreover, variability in soil properties can affect growth. Here, we aimed to identify drivers of small-scale growth variations with a pair-wise sampling approach at two ARD-affected orchards. We selected neighboring trees showing maximum differences in stem diameter growth. With this spatially explicit approach, large-scale heterogeneity in soil properties as a reason for differing growth was minimized. This design was applied to field plots differing in pre-cultures, i.e., comparing grass with *Tagetes patula,* the latter supposedly suppressing potential vectors of ARD*.* Various soil physical and chemical properties, the root phytoalexin content, and the bacterial and archaeal community composition were assessed. At one site, principal component analysis (PCA) separated neighbors with differing growth due to high particulate organic matter content, while no differences in soil physical properties, indicative of aeration differences or soil disturbances, could be detected. Elevated particulate organic matter content likely resulted from localized tree shredding. The worse-growing partners exhibited higher phytoalexin contents at the first site, which are general indicators of biotic stress and observed to increase in the presence of ARD. However, this was not associated with alterations of the rhizosphere bacterial and archaeal community composition as would be expected for ARD. At the second site, PCA showed no separation between tree groups, i.e., none of the measured variables could explain growth differences. Our work demonstrated that sampling neighboring trees with varying stem growth can identify co-occurring differences in related variables, some of which may reflect differences in ARD severity. Still, results were highly site-dependent and determined by the variables chosen for analysis.

## Introduction

Repeated planting of apple (*Malus domestica* Borkh.) in the same area leads to harmfully disturbed physiological and morphological reactions. This phenomenon, referred to as apple replant disease (ARD), is associated with strongly reduced shoot and root growth, as well as fruit yield and quality [1–3]. ARD is caused by biotic factors, as soil disinfection alleviates the effects [2,4,5]. Despite extensive research, no single organism has been identified as the causal agent, and it is most likely that the accumulation of certain potentially pathogenic biota is responsible [2,3,5,6]. ARD occurs in all major apple-growing regions in the world and persists in soils for decades [3,5–7]. ARD is most commonly counteracted by chemical soil fumigation. However, this approach involves the application of broad-spectrum biocides, which are potentially hazardous to the environment and/or human health [4–6]. On that account, various alternative approaches have been proposed, including breeding of less susceptible rootstock genotypes [8–10], biofumigation [11,12], modulation of root-associated microbiomes [13–16] or growing of pre-cultures prior to apple planting, like the nematode repellent *Tagetes patula* [6,8].

Previous studies have confirmed that ARD is distributed heterogeneously on a small scale, which may be attributed to the limited mobility of the ARD-causing agents [1,17]. These ARD patches can be spread in the soil by frequent clearing and tillage. Not only ARD can lead to variability in apple tree growth, but also the intrinsic heterogeneity of soil properties in the field. Soil formation results in distinct patterns that are determined by parental material, climate, relief, vegetation, human impact, etc. Because these factors differ at almost every spatial and temporal scale, abiotic soil factors vary considerably within fields [18,19]. As they exert important influences on plant growth, their in-field heterogeneity could be an additional reason for patchy growth [20].

Abiotic properties may exert direct effects on plant growth, but also indirect ones, as they are known to considerably control soil biota and shape their communities [21]. And as ARD is caused by biotic factors, chemical, and physical soil properties are often discussed as modulating factors of ARD [2,5,19]. Abiotic factors, therefore, not only directly influence tree growth but also have a major impact on the ARD-causing agents. Various studies have shown that multiple abiotic soil properties influence apple growth in ARD soil, with results often being contradictory [7,22–25].

The in-field severity of ARD is usually determined by measuring stem growth or plant height [24–26]. Besides the varying growth, ARD can be identified with ARD co-occurring stress indicators. The bacterial and archaeal community composition of the rhizosphere was shown to differ in ARD-affected and non-affected soils from the same site [27–29]. The biosynthesis of biphenyl and dibenzofuran phytoalexins has also been reported to increase in roots growing in ARD soil [30,31].

In this study, the sampling strategy was chosen to account for the spatial variability of ARD. We did so by pair-wise sampling of neighboring trees, which show maximum differences in growth as suggested by Tilston *et al.* [26]. The idea assumes that the heterogeneity of intrinsic soil properties is reduced at a small scale. As such, it is more likely to identify the factors that govern the variability in stem growth as neighboring trees presumably experienced highly similar growth conditions. We have chosen parameters which may indicate ARD severity (phytoalexins), and parameters related to the soil microbial dysbiosis due to ARD (bacterial and archaeal community composition). In addition, we included parameters reflecting differences in previous site management, such as uprooting frequency and soil cultivation (porosity, pore diameter, bulk density, water retention capacity), fertilizer application and organic matter input (soil pH, soil C, particulate organic matter), as well as intrinsic soil properties (texture). With this approach, we aimed to identify the drivers of small-scale growth variation using principal component analysis coupled with PERMANOVA.

The sampling strategy was implemented at two ARD-affected sites. In these orchards, the pairs varied in the culture that was sown prior to apple. For half of the pairs, the area was covered in grass for one vegetation period. For the other half, a *Tagetes* pre-culture was established. *Tagetes* has been shown to reduce the effects of ARD [32,33], which is attributed to its nematode-repelling properties [34,35] and its ability to alter microbial community composition [32].

In this study, we aim to improve the understanding of ARD heterogeneity under field conditions by addressing the following questions:

(1) What were the driving forces for growth differences of neighboring trees in ARD-affected apple orchards? (2) Did a *Tagetes* pre-culture have an effect on the variables driving growth differences? (3) Was the chosen pair-wise design a reasonable approach to identify ARD-related differences in the field?

## Materials & methods

### Study area

The field experiments were carried out at two different commercial apple orchards in northern Germany, which were kindly provided by the owners. Hereafter, the abbreviations “HS” (experimental site at 53.48213°N; 9.593103°E) and “BO” (experimental site at 53.57613°N; 11.13939°E) were used to denote the experimental sites.

Previous apple cultures were recorded for the two locations. The area at HS has been used for continuous apple growing since 1995. At BO, apples were cultivated until the 1990s, after which the location was used for agriculture for about 30 years before the establishment of the new plantation.

Both soils were shown to be affected by ARD. Corresponding biotests were conducted following the procedure described by Yim *et al.* [36], in which M26 apple plantlets were grown in soil taken from these sites. A reduction of plant biomass compared to plantlets in γ-irradiation sterilized soil was observed (S1 Table). Based on the observed growth reduction, HS was classified as “severely” and BO as “moderately severely” affected by ARD.

The texture for the sites was similar, both being categorized as very silty sand (according to KA5, [37]). Two pre-cultures were used by the growers: 1) grass pre-culture (treatment control) and 2) *Tagetes patula* pre-culture (treatment *Tagetes*). For the control treatment, grass was sown at HS (“Berliner Tiergarten Grassaat”, 60% *Lolium perenne*, 40% *Festuca rubra)* in spring 2020 and at BO (*Poa pratensis*) in spring 2019. For the *Tagetes* treatment, *Tagetes* “Nemamix” was sown (HS: Landhandel Dammann; BO: PROGRESS Agrar). In autumn of the respective years, the plants were chopped and incorporated into the soil. In March 2021, the apple variety “GS66” on rootstock M9 was planted at HS. At BO, the planting took place in November 2019 (“Golden Delicious”, mutant Reinders, on rootstock M9).

At both sites, two blocks were established. Each treatment was represented by one plot per block. Boundary trees were neglected to minimize influences of neighboring plots.

The growth parameters were determined from 30 trees (HS, 5 rows/ 6 trees) and 21 trees (BO, 7 rows/ 3 trees) per plot. The spacing between trees was 1 m within rows and 3.10 m between rows (HS), and 1 m and 3.50 m, respectively, for BO. HS operates as a conventional orchard that uses herbicides (glyphosate) for chemical weed control. In contrast, BO has been managed ecologically since 2020. As a result, only mechanical weed control has been applied since then (under-row hoeing machine “Krümler Ladurner”, Ladurner Karl J. & Co. OHG, Italy). Both sites use drip irrigation for watering.

In 2023, the annual mean temperature at the nearest weather stations was 10.61°C for BO and 10.66°C for HS. Precipitation levels in 2023 were recorded at 815.6 mm near BO and 1029.6 mm near HS. An overview of the site characteristics can also be found in the Supporting information (S2 Table).

### Sampling

Sampling took place in October (HS) and September 2023 (BO). The sampling design was based on a pair-wise comparison between trees that showed maximum variation in their stem growth (i.e., increase in stem diameter measured 20 cm above the graft). The difference between the stem diameter at planting and the last measurement before sampling (HS: April 2023; BO: September 2022) was determined to identify suitable pairs (see also S1 Fig). Each pair included one tree with higher growth and one with lower growth. In the following, the combination of treatment and growth will be referred to as a “tree group”. As such, four tree groups were defined per site: “Control-Better”, “Control-Worse”, “*Tagetes*-Better”, and “*Tagetes*-Worse”.

Each tree group consisted of five pairs. The pairs were evenly chosen between the two plots of the same treatment. Per tree, two samples were taken at a distance of 30 cm from the trunk, oriented perpendicular to the row (i.e., in direction of the neighboring rows). As one pair consists of two trees, four samples were collected for each pair.

**Fig 1:**
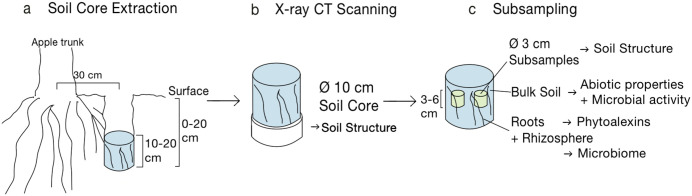
Schematic overview of the sampling procedure and further processing of the sampled soil cores.

To avoid the tillage-disturbed depth, the samples were extracted from a depth of 10–20 cm. Cylindrical soil cores (diameter (Ø) 10 cm, 10 cm height) were collected with a custom-made drill for undisturbed sampling (UGT GmbH, Germany). Sampling and further processing are schematically shown in Fig 1, where the first part (a) depicts extraction of the soil cores. The soil cores were encased in a stiff 3 mm acrylic glass sleeve and stored in plastic bags at ~5°C until scanning with X-ray CT.

One core could not be taken due to stones in the soil and was therefore missing in all subsequent analyses.

### X-ray CT scanning

X-ray computed tomography (CT) scanning to examine soil structure was performed with an X-ray microtomograph (X-TEK XTH 225, Nikon Metrology) having an Elmer-Perkin 1620 detector panel (1750 × 2000 pixels). After scanning the Ø 10 cm soil cores with 55 µm resolution (Fig 1b) two cylindrical subsamples (stiff PVC sleeves with Ø 3 cm, 3 cm height, wall thickness 2 mm) were extracted per core (Fig 1c). They were taken at 3–6 cm below the surface of the sample and scanned with 19 µm resolution. Because of the trade-off between sample size and resolution, the 3 cm cores were better suited to identify smaller-scale soil structures. In comparison, the 10 cm cores were used to identify larger constituents.

The CT scan settings for the larger cores were 160 kV, 390 µA, 0.7 mm Cu filter, 2748 projections, 500 ms exposure time, while for the 3 cm cores, 130 kV, 150 µA, 0.1 mm Cu filter, 2500 projections, 708 ms exposure time were used.

The projections per scan were reconstructed into a 3D tomogram having an 8-bit grayscale via a filtered back projection algorithm with the CT Pro 3D software (version XT 4.4.4, Nikon Metrology). During the 8-bit conversion, the contrast was optimized with a percentile stretching method. This method sets 0.5% of the darkest and brightest voxels to 0 and 255, respectively, and performs a linear stretching in between.

In total, 232 (78 Ø 10 cm; 154 Ø 3 cm) X-ray CT images were analyzed for this study. Besides the core that could not be taken in the field, one additional sample was excluded as the scan quality was insufficient. Stones, along with very loose substrate, also prevented subsampling on four occasions.

### Image processing and analysis

The grayscale images obtained from the X-ray CT scanning were processed using the Fiji bundle for ImageJ [38]. The cylindrical region of interest (ROI) was adjusted to be the largest cylinder that fits without including small fractures close to the sample wall. When cracks extended into the soil, the ROI was changed and deviated from the cylindrical shape.

The images were segmented into matrix, rocks, pores, and particulate organic matter (POM) using the random forest based LABKIT pixel classification plugin [39] in Fiji. For each combination of site and resolution, an individual classifier was trained using eight small sub-volumes. The pore structure was analyzed with respect to visible porosity and mean pore diameter according to the soil structure library protocol [40]. Mean pore diameter was determined with the local thickness method in Fiji, which is based on the maximum inscribed sphere method.

### Sampling of roots and soil

After scanning the Ø 10 cm cores and collecting the subsamples, the material remaining from the cores was used to determine various properties (Fig 1c).

Roots were visually assessed, and those most likely belonging to the apple trees (based on traits such as larger diameter and reddish coloring) were selected. From the chosen roots, tightly attached soil was gently removed with toothbrushes to detach the rhizosphere soil, which was collected in 2 mL reaction tubes and subsequently submitted to total microbial DNA extraction and microbial community analysis.

After brushing the roots, they were rinsed with water, dried on a paper towel, and immediately frozen in liquid nitrogen for phytoalexin extraction. Rhizosphere samples were stored at −20°C and roots at − 80 °C until further processing. The bulk soil from the cores was collected in plastic bags and stored at 4°C to assess abiotic soil properties and soil microbial activity.

### Characterization of abiotic soil properties

Soil pH was measured using a glass electrode (SevenDirect SD20 pH meter, Mettler Toledo, Germany) in a 0.01 M CaCl_2_ solution, with a soil-to-solution ratio of 1:2.5. Soil organic carbon (SOC) was determined using a CNS analyzer (Vario Max CN Element Analyzer, Elementar, Germany). Soil texture was assessed through automated particle size analysis using the “PARIO” device (METER Group, Munich, Germany).

### Measuring soil water retention

To determine soil water retention curves, one additional undisturbed sample (Ø 8 cm, 5 cm height) was collected per tree. These were taken within the row at a distance of 20 cm from the trunk. A simplified evaporation method coupled with the HYPROP measuring device (METER Group, München, Germany) was used to characterize the water retention curve of the samples. Before being placed in the apparatus the cores were slowly saturated. After the measurements, the samples were oven-dried at 105°C to determine bulk density.

### Extraction of total microbial DNA, amplicon sequencing, and sequence processing to determine bacterial and archaeal community composition

Total microbial DNA was extracted and subsequently purified from 0.5 g of each rhizosphere soil sample as described in Hauschild *et al.* [41].To analyze the bacterial and archaeal community composition, purified DNA extracts (ca. 10 ng µL^-1^) served as templates to amplify the variable V3-V4 region of the bacterial/archaeal *16S rRNA* gene using primers 341F/806R [42]. Amplicon sequencing, including PCR amplification, library preparation, and sequencing on an Illumina MiSeq v2 PE250, was performed at the sequencing service provider Novogene Co. (Munich, Germany) according to the company’s standard procedures.

Raw amplicon sequences were processed and classified to amplicon sequence variants (ASVs) as described previously [41]. Classification of ASVs using the SILVA database version 138.1 [43] resulted in 61,794 unique ASVs. Each ASV was annotated to the lowest possible rank curated in the database, and ASVs that were not classified at the phylum level or that were identified as chloroplast or mitochondria were removed from the dataset. ASVs that were unclassified at the genus level were labelled with the suffix _uc. Cleaned ASV, taxonomy, and metadata tables were imported to the “phyloseq” package (version 1.52.0, [44]). According to recommendation by Schloss [45], all subsequent analyses, except differential abundance testing, were performed on the rarefied dataset. Rarefaction was done across all samples based on the smallest sample size (20,930 sequence reads).

### Characterization of soil microbial activity

The soil dehydrogenase activity was selected as an indicator for soil microbial activity. It was measured following a standardized procedure as described in von Mersi and Schinner [46]. Briefly, 5 g of fresh and sieved (2 mm) soil (stored at 4° C) was treated with 5 mL of iodotetrazoliumchlorid solution (INT) and incubated for 4–6 h at 25° C. Afterwards, the released iodonitrotetrazolium formazan (INTF) was extracted with acetone. Adsorption was measured at 485 nm using a UV-VIS spectrophotometer (Lambda-35, Perkin Elmer, Rodgau, Germany).

### Phytoalexin extraction and analysis

The extraction and quantification of phytoalexins using gas chromatography- mass spectrometry (GC-MS) was performed according to well-established protocols published by Busnena *et al.* [30].

### Statistical analysis

Before performing any computations, the mean of the two replicate measurements per tree was calculated for each variable. This dataset was then used for all subsequent analyses.

For soil microbial activity, only one sample per tree was considered due to the time-consuming process. Additionally, for phytoalexin analyses, 5 pairs only contained one value per tree, as some samples were lost due to tube breakage during homogenization. In both cases, single samples per tree were treated as representative. One outlier at site HS was removed due to its phytoalexin content being approximately ten times higher than that of comparable samples, which was attributed to a measurement error. From the amplicon sequencing dataset, three samples from site HS were excluded from analyses because of their notably low number of sequences, as indicated in the rarefaction curves (S2 Fig).

Both sites were evaluated separately for all computations. In the following, a *p*-value < 0.05 is considered significant. All statistical analyses were conducted in R (R 4.4.2 for rhizosphere microbiome data, R 4.4.0 for all others; R Core Team [47]). The R-code for the analyses is available on GitHub (https://github.com/SophWach/LMM-and-PCA-for-pairwise-sampling.git).

Principal Component Analysis (PCA) was performed on the matrix-converted per-tree dataset that was subsequently normalized to eliminate scale differences. K-nearest neighbors (KNN) imputation was applied prior to transformation (package “VIM”, version 6.2.2, [48]) to estimate missing values and avoid excluding trees from the analysis. This concerned one value in the data set where phytoalexin data was missing for both tree samples. The princomp function (package “stats”, version 4.4.0, R Core Team) was used to conduct PCA. The results were visualized as score plots and loading plots using “factoextra” (version 1.0.7, [49]).

For PERMANOVA, the data was converted into a Euclidean distance matrix. To assess differences between tree groups, separate evaluations for treatment and growth state were necessary. Implementing our specific pair-wise design required differently defined permutation objects, as the two analyses demanded varying approaches to restricting permutations [50]. In the first design (whole-plot), the effect of treatment was tested. There, the treatments were shuffled between the pairs while the growth condition stayed fixed within each pair. In the split-plot design, the focus was on evaluating the growth conditions, where “better” and “worse” were shuffled within each pair.

After the definition of the restricted permutations, PERMANOVA was performed via the adonis2 function (package “vegan”, version 2.6.6.1, [51]).

As measure for the samples’ microbial diversity, Shannon-indices were calculated using the “vegan” package for each tree group. To display the microbial community composition, data were transformed to relative abundance, and the average relative abundance of the dominant bacterial taxa (relative abundance >1% in at least one tree group) was displayed in a heatmap using the “pheatmap” package (version 1.0.12, [52]). Bacterial/archaeal composition was analyzed in a principal coordinate analysis (PCoA) on the matrix-converted per-tree dataset using rarefied ASV count data. For PERMANOVA, the microbiome data was converted into a Bray-Curtis distance matrix, and implementation of the specific pair-wise-design was performed as described above for PCA. To identify bacterial/archaeal taxa with differential abundance among the tree groups, “ANCOM-BC2” (version 2.10.1, [53]) and “DeSeq2” (version 1.48.2, [54]) were applied to unrarefied count data on each taxonomic rank. Taxa were considered differentially abundant at an adjusted *p*-value (*p-adj)* <0.05. However, considering a prevalence >2, both tools did not reveal any taxa with differential abundance.

Linear Mixed Models (LMMs) were fit to assess the tree group effect on our determined variables (package “lme4”, version 1.1.35.3, [55]). The model defined the tree group factors (treatment and growth) as well as their interaction as fixed effects. To implement our chosen pair-wise design each pair was set to have a random intercept. Residual plots and statistical tests (using the “performance” package, version 0.12.0, [56]) were conducted to assess whether the LMM met the necessary assumptions for computation. For the majority, no strong violations were detected. When the tests detected non-normality or heteroscedasticity, a Generalized Linear Mixed Model (GLMM) was defined. The GLMM was fitted using the same structure as the LMM with the glmmTMB package (version 1.19, [57]), specifying a Gamma distribution with an identity link function. This model was also assessed for assumption violations using simulated residuals generated with “DHARMa” (version 0.4.6, [58]). For variables containing zeros instead of a Gamma distribution ziGamma (zero-inflated Gamma) was applied with a log-link function. *P*-values were generated from the model output using the “emmeans” package (version 1.10.2, [59]). Figures were produced with the package “ggplot2” (version 3.5.1, [60]).

In total, these models were fitted to 20 variables. Since the two sites were analyzed separately, this resulted in 34 LMMs and 6 GLMMs (4 Gamma, 2 ziGmma), making a total of 40 models included in this study.

## Results

### Assessment of sampling pre-conditions

The differences in stem growth between tree planting and our sampling varied significantly between the better- and worse-growing trees, independent of treatment and site (S3 Fig). This confirmed that the requirements for our pair-wise design were met at the time of sampling. Notably, the increase in stem growth was lower at site HS, which likely reflects the younger age of the HS trees.

Soil texture, as an intrinsic soil property, exhibited similar values across the tree groups at both sites (S4 Fig).

### Principal component analysis

**Fig 2:**
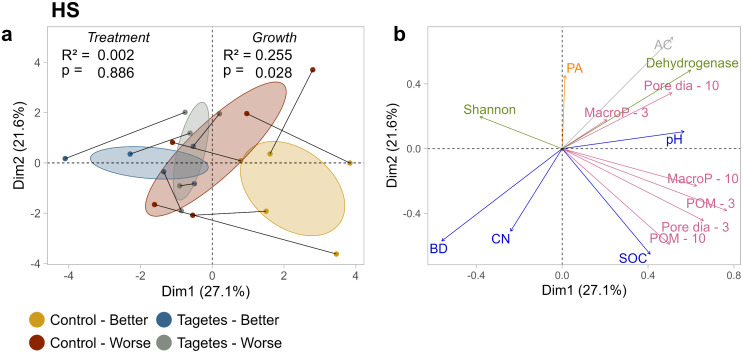
Score plots (a) and loadings plots (b) of the first two principal components (PC1 and PC2) for site HS. Values in parentheses next to each PC denote the percentage of variance explained by the respective principal component. In the score plots, measured values are represented as dots, while 95% confidence intervals are depicted as ellipses. Connected dots indicate paired data points. PERMANOVA results – separate for treatment level and growth status – are presented in the score plots, with R² values indicating the proportion of variance explained and *p*-values reflecting the strength of the observed patterns relative to random variation. In the loadings plots, different colors mark the variable groups soil structure, water retention, physico-chemical properties, microbiome, and phytoalexins. AC, air capacity; BD, bulk density; CN, ratio of C to N; SOC, soil organic carbon; MacroP – 10, macroporosity cores Ø 10 cm, −3 subsamples Ø 3 cm; POM, particulate organic matter; Pore dia, Pore diameter; PA, phytoalexins.

For site HS, distinct clusters of tree groups were visible in the PCA score plot (Fig 2a). The “Control–Better” group separated clearly along principal components 1 and 2 (PC1 and PC2). This directional trend can also be observed in the orientation of the connected tree pairs. As indicated by the corresponding loadings plot (Fig 2b), this was mainly driven by soil structure properties. “*Tagetes*-Better” was separated along PC1, where the pH value was located in Fig 2b. These patterns aligned with the PERMANOVA results, which showed a low *p*-value and high R² for growth status, suggesting that it explained a high portion of variance in this dataset.

**Fig 3:**
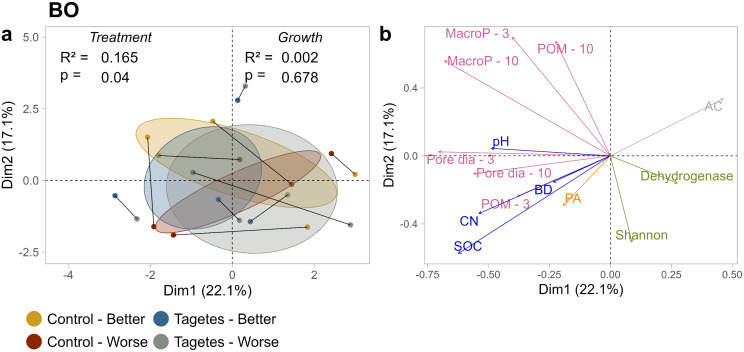
Score plots (a) and loadings plots (b) of the first two principal components (PC1 and PC2) for site BO. Values in parentheses next to each PC denote the percentage of variance explained by the respective principal component. In the score plots, measured values are represented as dots, while 95% confidence intervals are depicted as ellipses. Connected dots indicate paired data points. PERMANOVA results – separate for treatment level and growth status – are presented in the score plots, with R² values indicating the proportion of variance explained and *p*-values reflecting the strength of the observed patterns relative to random variation. In the loadings plots, different colors mark the variable groups soil structure, water retention, physico-chemical properties, microbiome, and phytoalexins. AC, air capacity; BD, bulk density; CN, ratio of C to N; SOC, soil organic carbon; MacroP – 10, macroporosity cores Ø 10 cm, −3 subsamples Ø 3 cm; POM, particulate organic matter; Pore dia, Pore diameter; PA, phytoalexins.

At site BO (Fig 3a), no clustering according to tree group was observed. This corresponded with a higher *p*-value and lower R^2^-value in the PERMANOVA for growth status. For treatment, R^2^ was slightly higher and the *p*-value lower, though this did not result in distinct treatment clusters. This suggests that neither treatment nor growth status strongly influenced the overall multivariate structure of the data. The connected partners of the tree pairs varied in orientation and proximity, indicating that the pairs were influenced by different combinations of the PCs and their associated loadings (Fig 3b).

### Soil properties as related to tree groups

**Fig 4:**
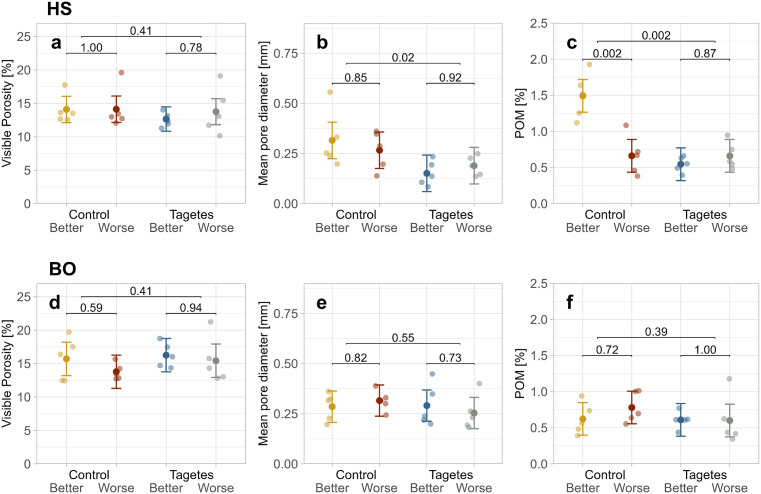
Jitter plots of soil structure properties measured in Ø 3 cm soil subsamples at site HS (a-c) and BO (d-f). Jittered points show the values per tree. The mean points represent the LMM predicted means, and the error bars indicate the 95% confidence intervals for these predictions. Bracket annotations connecting the 1st and 2nd as well as the 3rd and 4th group give the *p*-values that compare the different growth-status trees within the treatment. For comparisons between control and *Tagetes* treatments, all samples were included regardless of growth status; these *p*-values are shown on the upper brackets.

**Fig 5:**
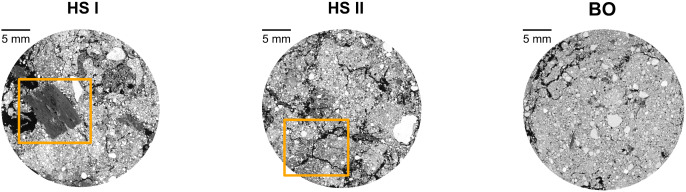
Example X-ray CT cross sections derived from Ø 3 cm soil subsamples from sites HS (HS I and HS II) and BO. The orange rectangles highlight characteristic features (HSI: POM, HSII: cracks).

At HS, the predicted mean for the particulate organic matter (POM) content in the “Control-Better” group was, at 1.5%, more than twice as high as that of the other tree groups (≈ 0.65%; Fig 4c). This resulted in a significant *p*-value for both the comparison between better- and worse-growing trees within the control group and the comparison between control and *Tagetes* treatments. In samples with high POM content, fibrous organic material was found in the CT cross sections (Fig 5, HSI). A similar trend was observed for the mean pore diameter, which was highest in “Control-Better” with 0.32 mm (Fig 4b). Yet, in this case, “Control-Worse” was also increased compared to the *Tagetes* groups (≈ 0.17 mm), resulting in a significant difference between treatments. This increase in mean pore diameter was accompanied by a greater formation of cracks (Fig 5, HSII).

At site BO, however, the analysis of soil subsamples (Ø 3 cm) revealed no significant differences in soil structure variables between tree groups (Fig 4d-f). The corresponding results for the coarser resolution (soil cores Ø 10 cm) led to similar results (S5 Fig).

**Fig 6:**
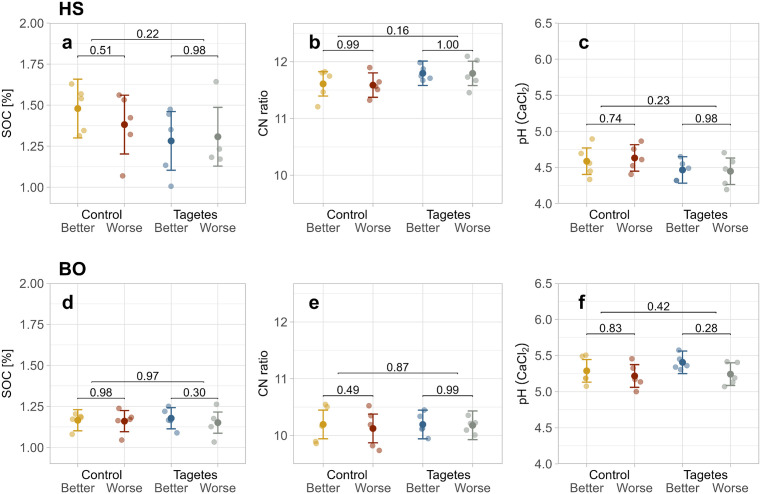
Jitter plots of physico-chemical soil properties at site HS (a-c) and BO(d-f). Jittered points show the values per tree. The mean points represent the LMM predicted means, and the error bars indicate the 95% confidence intervals for these predictions. Bracket annotations connecting the 1st and 2nd as well as the 3rd and 4th group give the *p*-values that compare the different growth-status trees within the treatment. For comparisons between control and *Tagetes* treatments, all samples were included regardless of growth status; these *p*-values are shown on the upper brackets.

At HS, the highest predicted mean of soil organic carbon content (SOC) was observed in the “Control–Better” group (1.48%; Fig 6a). However, the distribution of individual tree values was similar across groups, with the lowest and highest mean differing by only 0.2%. At BO, SOC did not differ between tree groups (Fig 6d). For the CN ratio, no significant differences were identified, though at HS, the *Tagetes* groups showed slightly higher values (≈ 11.8) compared to the control (≈ 11.6; Fig 6b).

No significant differences in pH were observed between the tree groups at either site (Fig 6c, 6f). However, a slight effect of *Tagetes* was noted. At HS, pre-culture *Tagetes* was associated with a lower pH (*Tagetes* ≈ 4.46, control ≈ 4.61), whereas at BO, the *Tagetes* groups showed a slight increase in pH (*Tagetes* ≈ 5.33, control ≈ 5.25).

Air capacity, available water capacity, and bulk density showed no significant differences among tree groups for both sites (S6 Fig).

### Bacterial and archaeal community composition as related to tree groups

**Fig 7:**
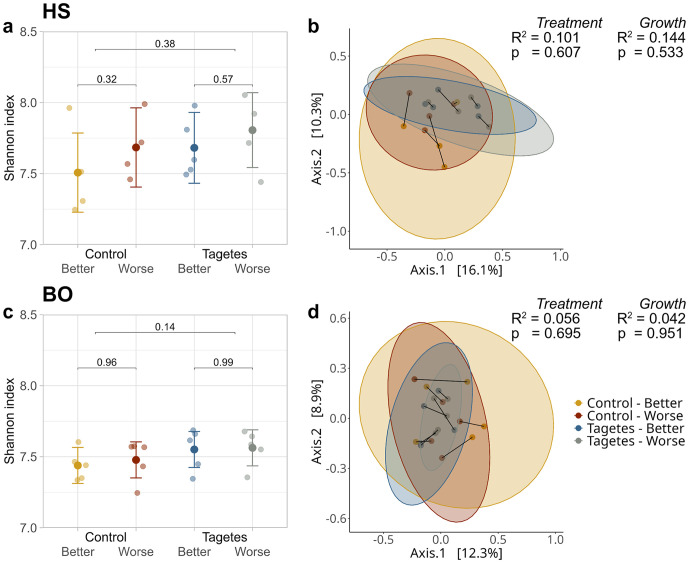
Jitter plots of bacterial/archaeal Shannon-indices as a measure for rhizosphere diversity at site HS (a) and BO (c), and principal coordinate analysis (PCoA) of bacterial/archaeal rhizosphere community composition for the same sites (HS-b; BO-d). In the jitter plots, jittered points show the values per tree. The mean points represent the LMM predicted means, and the error bars indicate the 95% confidence intervals for these predictions. Bracket annotations connecting the 1st and 2nd as well as the 3rd and 4th group give the *p*-values that compare the different growth-status trees within the treatment. For comparisons between control and *Tagetes* treatments, all samples were included regardless of growth status; these *p*-values are shown on the upper brackets. In the PCoA values in parentheses next to each PCoA-axis denote the percentage of variance. Samples are represented as dots, while 95% confidence intervals are depicted as ellipses. Connected dots indicate paired data points. PERMANOVA results – separate for treatment and growth status – are presented in the score plots, with R² values indicating the proportion of variance explained and *p*-values reflecting the strength of the observed patterns relative to random variation.

The predicted mean of the Shannon indices at HS ranged from 7.5 in the “Control–Better” group to 7.8 in “*Tagetes*–Worse” (Fig 7a), while at BO they were approximately 7.5 for all tree groups (Fig 7c). In both sites, no statistically significant differences occurred, indicating a similar bacterial/archaeal diversity in the rhizosphere.

PCoA revealed no clustering according to tree group at site HS (Fig 7b). This was confirmed by PERMANOVA, in which growth as well as treatment yielded a high *p*-value and low R^2^-value, suggesting that only a small proportion of variance could be explained by these factors. Very similar results were obtained for BO, where no clustering or significant effects were observed (Fig 7d). At both sites, the connected partners did not align in one direction along the PCoA-axis, suggesting no distinct bacterial/archaeal rhizosphere community compositions between pairs. Interestingly, the 95% confidence intervals of the control groups were notably bigger compared to those of the *Tagetes* groups, suggesting more similarity among *Tagetes* rhizosphere communities.

**Fig 8:**
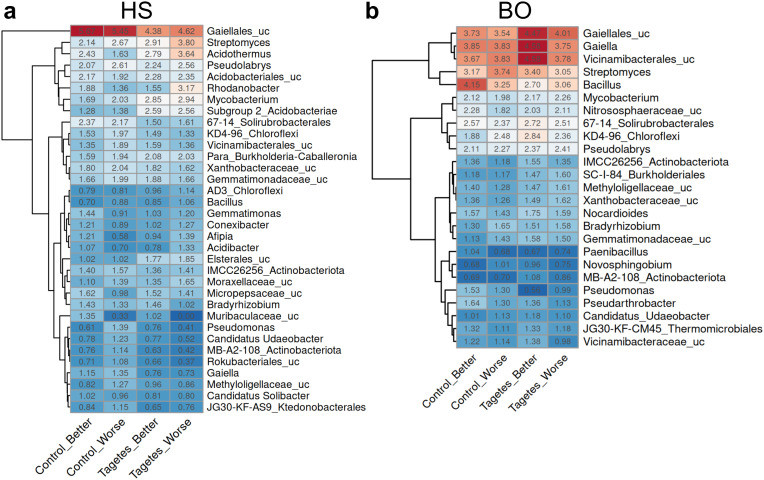
Heatmaps displaying the average relative abundance (n = 5) of dominant bacterial/archaeal taxa in the rhizosphere of apple trees from site HS (a) and BO (b). Taxa with a relative abundance >1% in at least one group are depicted. Abundances are color-coded from red (more abundant) to blue (less abundant). Differential abundance testing using ANCOM-BC2 and DeSeq2 did not reveal significant differences (p-adj < 0.05, Benjamini-Hochberg correction) for treatment or growth status for any taxon.

At both sites, the relative abundance analysis revealed some small differences in the dominant bacterial/archaeal taxa between tree groups, though none of them were significant (Fig 8).

In HS rhizospheres, the relative abundance of the most dominant group (unclassified *Gaiellales*) was notably higher (≈ 1%) in the control groups compared to the *Tagetes* groups (Fig 8a). Other dominant taxa *Streptomyces*, *Acidothermus*, and *Rhodanobacter* showed differing relative abundance depending on the *Tagetes* group, with higher levels (≈ 0.9%) observed in the partners with worse growth.

In BO rhizospheres, *Gaiella, Strepomyces, Bacillus*, unclassified *Gaiellales*, and unclassified *Vicinamibatcerales* were the dominating taxa (relative abundance > 3%; Fig 8b). From these dominant taxa, *Gaiella,* unclassified *Gaiellales*, and unclassified *Vicinamibatcerales* had a slightly higher relative abundance (≈ 0.5%) in the *Tagetes* groups than in the control groups. In contrast, *Bacillus* showed a small decrease (≈ 0.8%) in *Tagetes* compared to the control.

Also, dehydrogenase activity as a measure for soil microbial activity showed little to no variation with tree group for both sites (S7 Fig).

### Root phytoalexin content as related to tree groups

**Fig 9:**
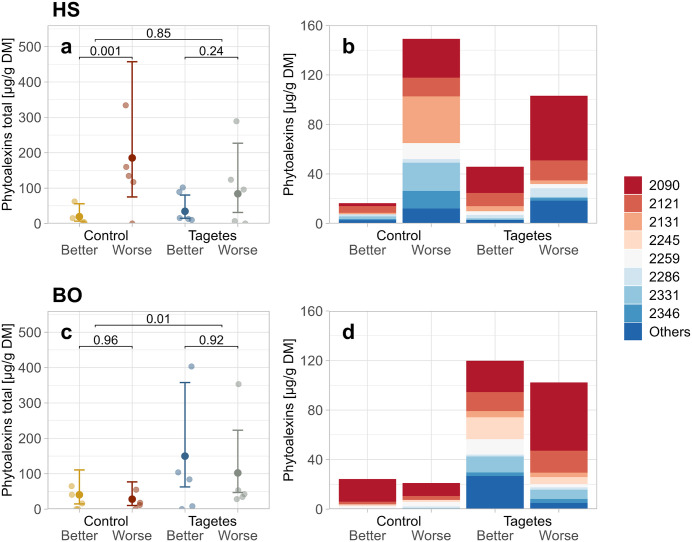
Jitter plots of phytoalexin (PA) content at site HS (a) and BO (c), and stacked bar plots of individual PA compounds for the same sites (HS–b; BO–d). In the jitter plots, jittered points show the values per tree. The mean points represent the LMM predicted means, and the error bars indicate the 95% confidence intervals for these predictions. Bracket annotations connecting the 1st and 2nd as well as the 3rd and 4th group give the p-values that compare the different growth-status trees within the treatment. For comparisons between control and *Tagetes* treatments, all samples were included regardless of growth status; these *p*-values are shown on the upper brackets. Compounds are indicated in the order of increasing Retention Index: 2090, aucuparin; 2121, noraucuparin; 2131, 2-hydroxy-4-methoxydibenzofuran; 2245, methoxyeriobofuran isomer 1; 2259, noreriobofuran; 2286, hydroxynoreriobofuran isomer 1; 2331, hydroxyeriobofuran isomer 2; 2346 eriobofuran isomer 2; Others: individual compounds with contents below 90 µg/g DM, based on sum of all sampled trees per site. Numerical values for all individual compounds can be found in the Supporting information (S3 Table).

At HS, worse-growing trees exhibited higher total phytoalexin contents than better-growing trees. In the control, their mean content was nearly ten times higher (19 and 185 µg/g DM-dry matter), leading to a *p*-value < 0.05 (Fig 9a). In *Tagetes*, it was still over twice as high in the worse-growing trees (35 and 84 µg/g DM), though this effect was not statistically significant. The composition of individual phytoalexins in the *Tagetes* groups was similar between better- and worse-growing trees, with aucuparin (Retention Index = 2090) being the dominant compound (Fig 9b, S3 Table). Notably, the aucuparin content was higher in the worse-growing trees, which largely accounted for the overall increase in this group. The composition in the worse group of the control treatment was similar to that of the corresponding growth group in the *Tagetes* treatment. However, methoxyeriobofuran isomer 1 (2245) showed the highest content under these conditions. The increased presence of this compound, together with aucuparin (2090) and hydroxyeriobofuran isomer 2 (2331), was the main driver of the general elevation in “Control-Worse”.

At BO, however, the increased phytoalexin values were driven by treatment rather than growth (Fig 9c). Contents were significantly higher in the *Tagetes* groups (≈ 126 µg/g DM) compared to the control groups (≈ 34 µg/g DM; Fig 9c). No significant differences were observed between better- and worse-growing trees within either treatment. Among the individual phytoalexins (Fig 9d, S3 Table), aucuparin (2090) again was the dominant phytoalexin across all subsets, with notably higher contents in the *Tagetes* groups. This led to the overall increase in those groups, together with a higher content of noraucuparin (2121) and a generally greater variety of phytoalexin compounds.

## Discussion

### Tree growth in relation to key variables

The higher POM content for the tree group “Control-Better” at site HS was particularly noticeable in the soil structure results. This also led to the clear separation of the “Control-Better” group in the PCA of HS (Fig 2a). A visual assessment of the X-ray CT images showed fibrous organic material in these samples (Fig 5, image HS II). Consultation with the orchard owner revealed that these were probably residues from old apple trees. During the previous apple planting (2011–2019), major damage due to voles occurred, and the affected trees were then shredded on the spot. Our observations may have been remnants of this procedure.

The input of additional organic matter provides available carbon. This is consistent with our data (Fig 6a), where we observed a slight increase in SOC for the “Control-Better” group. The additional carbon could boost the soil biological activity and thus lead to a higher diversity of beneficial microorganisms [61]. As such, it can increase soil suppressiveness and stimulate plant growth [6,7, and citations therein]. This is also why the use of various organic amendments has been suggested as a countermeasure to ARD. However, most studies report no or insufficient reduction of ARD through organic matter addition [1,7]. For bacterial/archaeal community composition analysis, the rhizosphere and not the area around the incorporated carbon source (shredded wood particles) was sampled. Therefore, its effect is not expected to be represented in this data, nor in the data for microbial soil activity, which was analyzed in the whole bulk soil fraction. This increase in POM in “Control-Better” was not observed in the *Tagetes* groups, which is not necessarily unexpected, as the input of shredded trees is highly localized. Given its site-specific nature, it is not surprising that this effect was absent at BO.

At site HS, the root phytoalexin contents exhibited the expected trend: worse-growing partners showed increased phytoalexin production, suggesting that they were subjected to biotic stress or infection [62], and citations therein]. Based on our initial assumption that the growth differences were governed by ARD, the worse-growing trees were considered more affected. Likely, as a result, they experienced greater stress, leading to elevated phytoalexin contents. This observation aligns with several studies reporting that phytoalexin production in roots was stimulated when apple plants were grown in ARD-affected soils [61,63,64].

Several phytoalexins with elevated contents in roots of the worse-growing trees have also been detected in previous research on the effects of ARD. These include reports on 2-hydroxy-4-methoxydibenzofuran (2131) [63], hydroxyeriobofuran isomer 2 (2331) [64], and aucuparin (2090) [31], which showed higher contents in roots grown in ARD soil. But it should be noted that these studies used young *in vitro* propagated M26 plantlets in controlled greenhouse experiments instead of commercial M9 rootstocks in field trials.

The three abovementioned phytoalexin compounds were also detected in the better-growing tree groups but at lower contents, indicating that they were affected by ARD as well, though to a lesser extent than in the worse-growing trees. This aligns with the expectations, as all trees were grown in ARD soil.

Interestingly, although biotests confirmed that site BO was affected by ARD, no growth-state-dependent phytoalexin response was observed. Notably, ARD severity in the biotest was rated as “medium-severe” at BO and “severe” at HS, suggesting that BO was generally less affected by ARD, which may have resulted in a weaker phytoalexin response. Another possible explanation is that the trees at BO had been planted a year earlier than those at HS, potentially having already passed the stage during which ARD symptoms were most severe. According to Hoestra [1] ARD symptoms tend to be most pronounced during the first year after planting. Over time, trees can overcome ARD by extending their roots into less-affected soil. While growth eventually normalizes, early variations in development remain visible. Moreover, with prolonged exposure, trees may acclimate to the growth conditions, leading to a reduced defense response. This phenomenon was demonstrated in a pot experiment by Siefen *et al.* [64], where phytoalexin contents in roots grown in ARD-affected soil declined over time. Generally, phytoalexins are not specific to ARD and have been reported to accumulate in apple roots in response to various stress factors [16,65,66]. In this study, the assumption that the observed responses are ARD-related is reasonable, as the sites are known to be affected by ARD. However, it is important to acknowledge that various other stress factors, beyond ARD, could also induce phytoalexin accumulation. Note that fungi, nematodes and root endophytes were not investigated in this study.

### Impact of *Tagetes* pre-culture in relation to key variables

Before commenting on the pre-cultivation effect, it should be noted that for both sites, a non-uniform distribution of the *Tagetes* cover was reported. The overall impact of *Tagetes* might have been less pronounced than intended, potentially reducing the observed differences to the control areas with grass pre-culture. Also, the *Tagetes* “Nemamix” is not a standardized seed mixture. As it was sourced from two different producers for the two sites, its exact composition may have varied.

Regarding abiotic soil properties, the most notable difference between control and *Tagetes* was the larger mean pore diameter in the control groups at site HS. For an explanation, X-ray CT images were again visually assessed. They revealed cracks that occurred in the control groups of HS, mainly in “Control-Better”, but not in the *Tagetes* groups (Fig 5, image HSI). These differences are likely due to variations in water balance, despite both areas being irrigated equally. Possible explanations for this include differences in microclimate (more shading in *Tagetes* pre-culture) or higher water requirement of the control treatments’ grass vegetation. However, the magnitude of desiccation cracks was still surprising, as the clay content—typically associated with the capacity of soils to shrink and swell [67]—is very low. Also, this effect could not be confirmed at site BO.

Additionally, a slight *Tagetes* effect on pH was observed, though it showed opposite trends at the two sites. To investigate this inconsistency, grass and *Tagetes* were cultivated in a side experiment under controlled conditions. The results suggest that differences in nitrogen demand and preferences for specific inorganic nitrogen forms between the two species could explain the divergent pH effects observed at site HS. This interpretation aligns with findings by Neina [68], who identified plant uptake of specific nitrogen forms as a primary driver of rhizosphere pH changes. Further details are provided in the Supporting information (S8 Fig). Although a very slight effect, it probably led to the separation of the “*Tagetes*-Better” tree group in the PCA of site HS (Fig 2a).

At BO, instead of a growth effect, a treatment effect regarding phytoalexin content was observed. Also, at HS, the better-growing trees of the *Tagetes* group showed elevated contents compared to “Control-Better”. These findings indicate that trees following *Tagetes* pre-culture experienced biotic stress at the time of sampling. However, the specific cause of this stress remains unclear, as the bacterial and archaeal microbial community composition in the rhizosphere provided no conclusive explanation.

### Unexpected absence of difference between key variables and tree groups

Altogether, few of the analyzed variables showed differences between neighboring trees with varying growth states. However, this would not necessarily be anticipated for many of them. E.g., for pH, Aggelopoulou *et al.* [69] found only a small spatial variability in a 0.8 ha apple orchard at a depth of 0–30 cm.

This lack of difference, though, is unexpected for the rhizosphere bacterial and archaeal community composition. When worse growth is attributed to ARD, a change would be assumed, as in most studies, ARD is accompanied by a shift in the rhizosphere microbiome, including bacterial and archaeal communities [2, and citations therein].

In our study, we found no significant differences in rhizosphere bacterial and archaeal communities across tree groups – independent of site, treatment, or growth status. Instead, we observed a consistent presence of taxa typically observed in the rhizosphere [28,29,70,71].

However, studies evaluating the effect of ARD on the rhizosphere microbiome usually compared ARD-affected soils to non-ARD reference soils, such as soils never planted with apple or soils that have been disinfected through γ-irradiation or fumigation. The more subtle nature of our comparison, focusing on trees with differing growth status on the same site, can provide a conclusive explanation for why such pronounced microbial differences were not observed. Nonetheless, Tilston *et al.* [26], who also established a pair-wise sampling design, reported significant differential abundance in approximately 1% of bacterial operational taxonomic units (OTUs) between tree pairs. However, their comparison involved ARD symptomatic and healthy trees, suggesting a more pronounced difference in growth status than in our study.

Although we did not detect differences in the rhizosphere bacterial and archaeal communities, the clear divergence in phytoalexin contents between tree groups suggests that underlying microbial shifts may still be occurring. Since phytoalexin production is largely induced by plant–microbe interactions, such changes in secondary metabolite profiles are often associated with alterations in the rhizosphere microbial community [30,63,72]. Additionally, selected microbes are capable of utilizing phytoalexins as a source of carbon [73]. Trees with elevated phytoalexin contents were expected to contain higher abundances of such microbes in their rhizosphere. At the same time, root-associated microbiota were shown to be inhibited by phytoalexins [74], indicating a close and dynamic relationship between phytoalexin profiles and microbial community composition.

Independent of varying phytoalexin contents, the absence of a *Tagetes* effect was also unexpected. Several studies have reported shifts in the rhizosphere microbiome following *Tagetes* cultivation [32,41,75]. One possible explanation for the absence of a *Tagetes* effect is the time gap between pre-culture and sampling. Previous studies that tested *Tagetes* in ARD-affected soils were conducted in the greenhouse [41] or in tree nursery field trials [32], and samples were analyzed only a few weeks after the treatments. This suggests that the *Tagetes* treatment did not induce a lasting modulation of the rhizosphere microbiome in apple orchards. Including additional sampling time points, particularly one in the year following *Tagetes* pre-culture, would have been valuable to capture possible immediate effects.

Also, in contrast to other studies [24,76,77], the control plots were not left bare but were instead covered with grass, which was later incorporated into the soil. Although the used grass species are not typical catch crops, their integration likely influenced the rhizosphere microbiome. The incorporation of biomass potentially contributed to a greater similarity between treatments by increasing the available carbon and altering resource availability, potentially favoring copiotrophic microorganisms [41,78]. Likely, the amendment of *Tagetes* and grass are more similar in shaping the rhizosphere microbiome than would have been expected from a site without a plant cover as control.

Also, most of the studies cited above were conducted under greenhouse conditions. Field sampling, and more so sampling in a commercial orchard, involves a wider range of environmental variables, which makes it more challenging to directly apply greenhouse findings to field settings.

Additionally, studies have focused on different apple genotypes. Comparing them can be difficult, as they recruit different rhizosphere microbiota and induce different plant reactions [64,72,79].

Besides the rhizosphere bacterial and archaeal communities, it is also unexpected that the soil microbial activity showed no difference in relation to the growth status of the trees. When worse growth is attributed to a stronger influence of ARD, it would be assumed to be accompanied by lower microbial activity, as soil productivity typically declines under ARD conditions. For example, Zydlik *et al.* [80] demonstrated that dehydrogenase activity was significantly lower in ARD-affected compared to non-ARD soil. But it is important to note that their analysis focused on rhizosphere soil. This suggests that ARD-related effects on microbial activity may be localized to the rhizosphere and thus not detectable in our bulk soil samples.

### Opportunities for optimizing the sampling approach

The core principle of our study design was to examine the “typical” orchard environment. While this approach reflects real-world conditions, it presents challenges in achieving optimal observation conditions, such as maximizing stem growth differences between neighboring trees or minimizing external influencing factors.

As a result, the relationships between the variables were not all clearly understood. Particularly, the stress-inducing factor leading to elevated phytoalexin content remained unidentified.

To gain a more comprehensive understanding, it might have been beneficial to expand the range of variables analyzed. For example, by including a broader spectrum of the rhizosphere microbiome, such as oomycetes and fungi, which are frequently reported to be pathogenic towards apple [2,81]*.* Analyzing root endophytes could also have provided valuable insights, as pathogenic root-endophytic fungi have been identified as key contributors to reduced apple plant growth in ARD-affected soils [82,83]. Furthermore, investigating soil fauna may have offered additional perspectives. Free-living nematodes, for instance, have been proposed as vehicles for ARD-associated pathogenic microbes [33, and citations therein]. Also, collembola are affected by ARD, showing a significant preference for colonizing non-ARD soils over ARD soils [84].

It would also have been valuable to examine the microbiome not only from a taxonomic perspective using short-read amplicon sequencing, but also at the functional level. It is well-known that bacteria with identical *16S rRNA* gene fragments, resulting in identical taxonomic annotation, can have diverse functional genes, such as those encoding different plant-beneficial functions [85]. Also, functional traits of microbiota are often strain-specific, going below the resolution provided by *16S rRNA* gene amplicon sequencing, and can vary for the same strain under different environmental conditions [86,87]. Functional analysis could have been achieved using cultivation-independent methods like metatranscriptomics or by cultivation-dependent isolation and characterization of rhizosphere microbiota [88,89].

To enhance the effect of *Tagetes* pre-culture, it might be beneficial to grow *Tagetes* as an undersown crop. This approach was tested in an ARD-affected orchard in China and revealed altered soil-physico-chemical properties, soil enzymatic activities, and bacterial community composition [75]. Further, the authors showed that *Tagetes* intercropping enhanced starch and sucrose metabolic pathways compared to clean tillage, but they did not assess effects on apple tree growth. Although permanent undersown *Tagetes* could be a promising approach for the sites investigated in this study, it is generally avoided in practice due to increased vole activity observed in such systems.

Furthermore, the selection of pairs could have been optimized to ensure that neighboring trees with the greatest contrast in growth performance were included. This might have been achieved by incorporating a broader range of growth indicators in the evaluation, such as yield or belowground biomass.

## Conclusion

Overall, our work demonstrated that sampling neighboring trees with varying stem growth can identify co-occurring differences in related variables.

For one site (HS), some clear dissimilarities in abiotic soil properties (POM) and secondary stress indicators (phytoalexins) were found between trees of unequal growth status. The increase in phytoalexins in worse-growing trees was attributed to ARD influence, serving as a strong indicator that growth differences at this ARD-affected site were most consistent with an influence of ARD. Despite the observed dissimilarities, many connections remain unclear—particularly why the increase in phytoalexins was not accompanied by changes in the rhizosphere bacterial and archaeal community composition. The underlying cause of stress indicated by the phytoalexin increase remains unknown. Gaining clarity on this would have been valuable, as it could have provided insights into the still unknown ARD-causing mechanism. The *Tagetes* pre-culture did not affect the variables driving growth differences at HS. At the other site (BO), none of the selected variables explained the differing tree growth. In terms of pre-culture, *Tagetes* led to an increased phytoalexin content, indicating biotic stress in these trees. The underlying reasons for this effect remain unclear.

To conclude, growth differences may represent the combined effect of ARD alongside other unmeasured biotic factors. Successful identification of growth-influencing circumstances seems to be highly dependent on the variables chosen for analysis and the site. Still, pair-wise sampling can offer an approach for evaluating small-scale processes (like ARD) where effects are often masked by the vast in-field heterogeneity of various factors.

## Supporting information

S1 Fig Schematic overview of the sampled orchards HS and BO. (DOCX)

S2 Fig Rarefaction curves based on ASVs derived from amplicon sequencing of the 16S rRNA gene of microbial community DNA from rhizosphere soil for both sites (BO and HS). (DOCX)

S3 Fig Jitter plots of the increase in stem diameter (from planting until sampling) at site HS (a) and BO (b). (DOCX)

S4 Fig Jitter plots of soil texture at site HS (a-c) and BO (d-f). (DOCX)

S5 Fig Jitter plots of soil structure properties measured in Ø 10 cm soil cores at site BO (a-c) and HS (d-f). (DOCX)

S6 Fig Jitter plots of air capacity (pF range 0–1.8), available water capacity (pF range 1.8–4.2), and bulk density at site HS (a-c) and BO (d-f). (DOCX)

S7 Fig Jitter plots of dehydrogenase activity at site HS (a) and BO (b). (DOCX)

S8 Fig Jitter plots of soil pH measured in bulk and rhizosphere soil from columns planted with grass or *Tagetes patula.* (DOCX)

S1 Table Biotest results as an indicator for ARD severity. (DOCX)

S2 Table Soil properties, ARD severity, and cultivation details for both study sites. (DOCX)

S3 Table Mean of single phytoalexins per tree group. (DOCX)
