# Supplementary material for: Detecting apple replant disease in the field – deciphering reasons for local growth depression
Source: PLoS One. 2026 Apr 21;21(4):e0345851. doi: 10.1371/journal.pone.0345851 (PMC13098943; doi:10.1371/journal.pone.0345851)
Supplement: S1 Fig — (DOCX) [file pone.0345851.s001.docx]

**
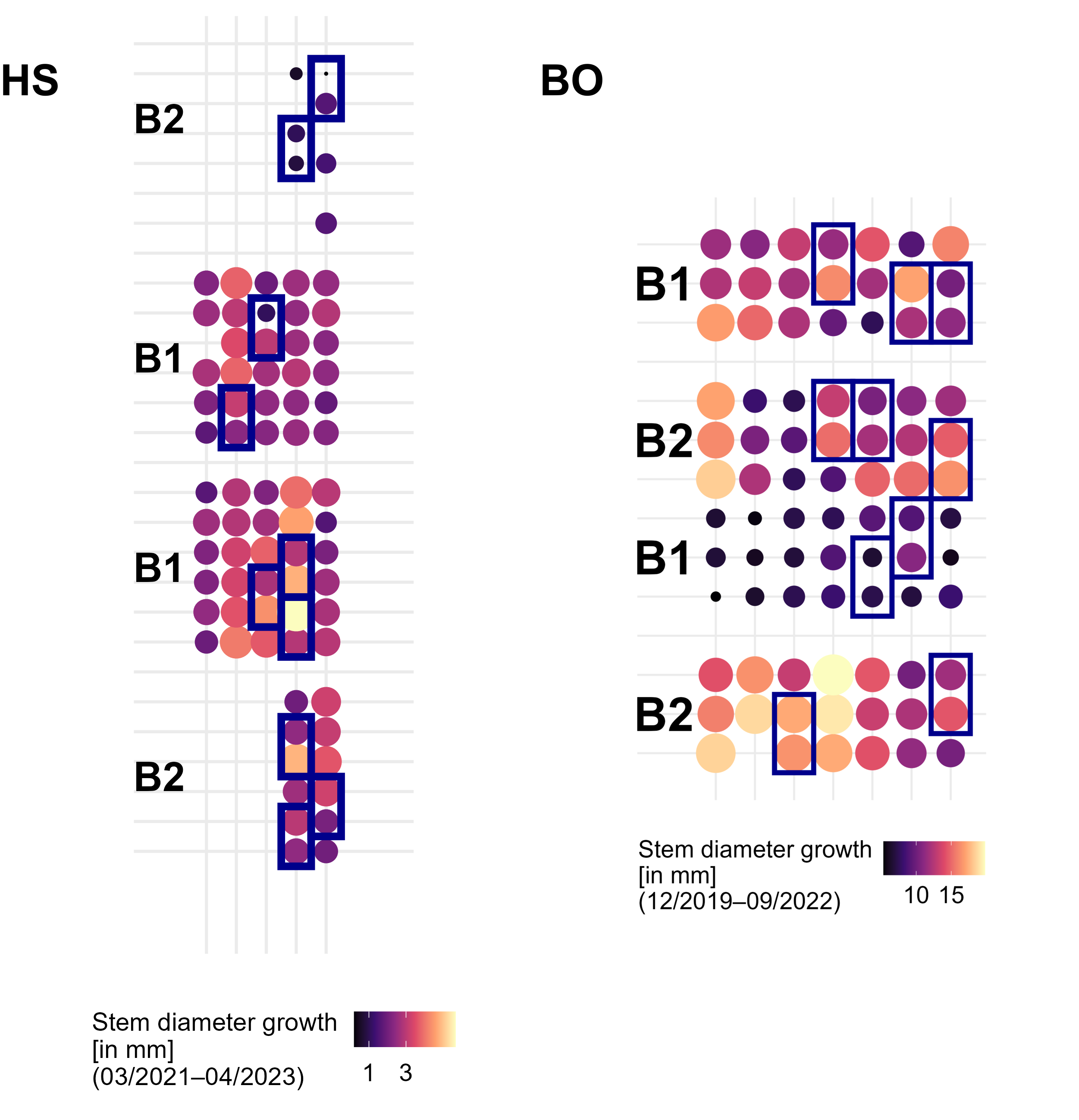
**

**S1 Fig. Schematic overview of the sampled orchards HS and BO.** B1 (Block 1) and B2 denote the two pre-culture treatments: B1 = grass pre-culture (“control”), and B2 = *Tagetes patula* pre-culture (“*Tagetes*”). Dots represent individual trees; dot size and color indicate stem diameter growth from planting to sampling (large/orange = high, small/violet = low). Blue boxes highlight the sampled pairs. Gaps between blocks indicate additional trees not included in the figure
