# Supplementary material for: Detecting apple replant disease in the field – deciphering reasons for local growth depression
Source: PLoS One. 2026 Apr 21;21(4):e0345851. doi: 10.1371/journal.pone.0345851 (PMC13098943; doi:10.1371/journal.pone.0345851)
Supplement: S2 Fig — (DOCX) [file pone.0345851.s002.docx]

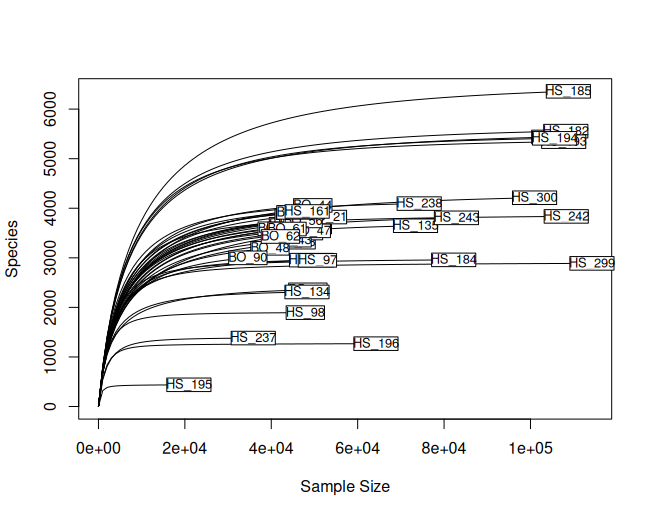


**S2 Fig. Rarefaction curves based on ASVs derived from amplicon sequencing of the *16S rRNA* gene of microbial community DNA from rhizosphere soil for both sites (BO and HS).**
