# Supplementary material for: Detecting apple replant disease in the field – deciphering reasons for local growth depression
Source: PLoS One. 2026 Apr 21;21(4):e0345851. doi: 10.1371/journal.pone.0345851 (PMC13098943; doi:10.1371/journal.pone.0345851)
Supplement: S4 Fig — (DOCX) [file pone.0345851.s004.docx]

**
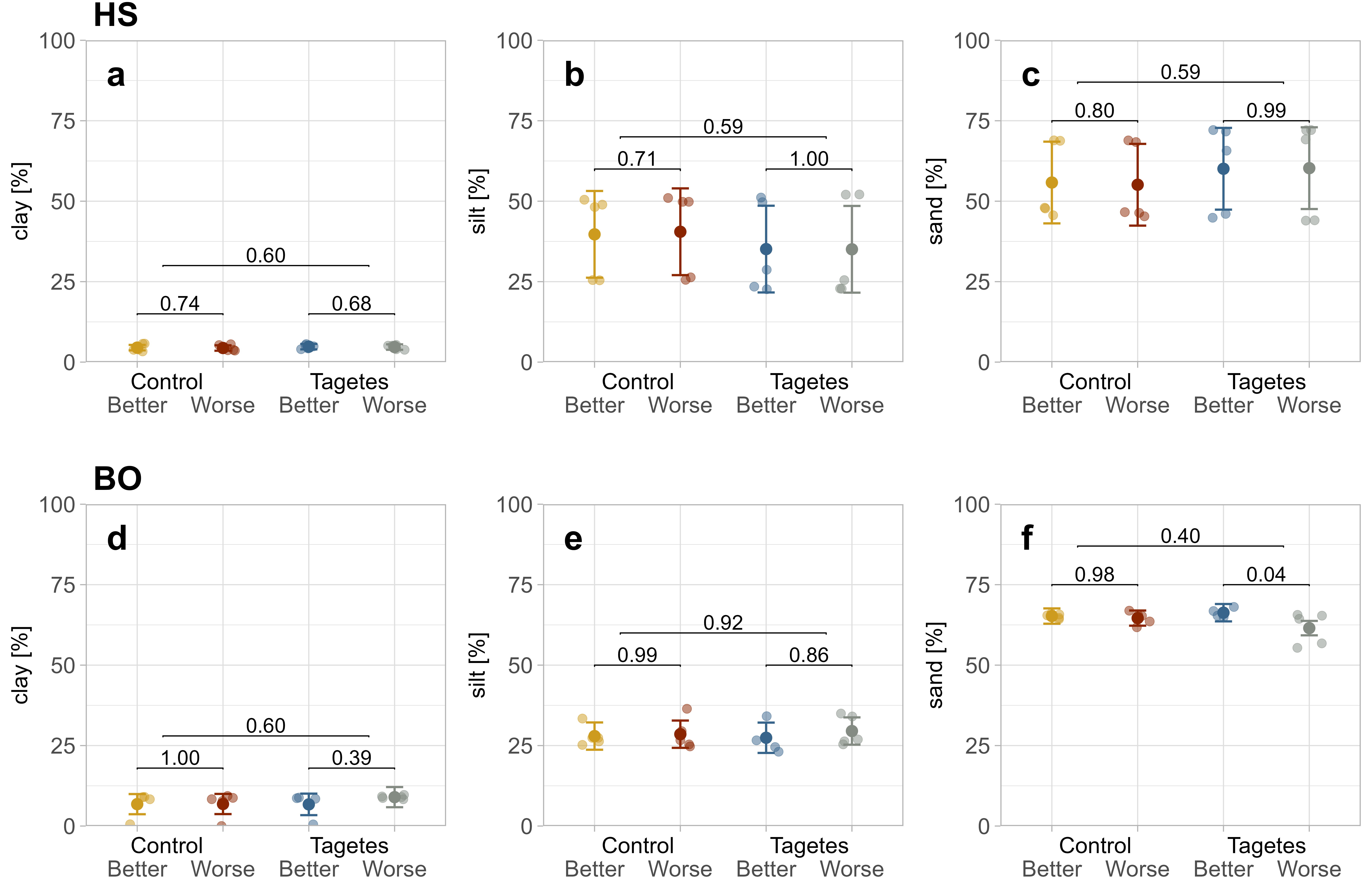
**

**S4 Fig. Jitter plots of soil texture at site HS (a-c) and BO (d-f).** Jittered points show the values per tree. The mean points represent the LMM predicted means, and the error bars indicate the 95% confidence intervals for these predictions. Bracket annotations connecting the 1st and 2nd as well as the 3rd and 4th group give the *p*-value s that compare the different growth-status trees within the treatment. For comparisons between control and *Tagetes* treatments, all samples were included regardless of growth status; these *p*-values are shown on the upper brackets.
