# Supplementary material for: Detecting apple replant disease in the field – deciphering reasons for local growth depression
Source: PLoS One. 2026 Apr 21;21(4):e0345851. doi: 10.1371/journal.pone.0345851 (PMC13098943; doi:10.1371/journal.pone.0345851)
Supplement: S8 Fig — (DOCX) [file pone.0345851.s008.docx]

**
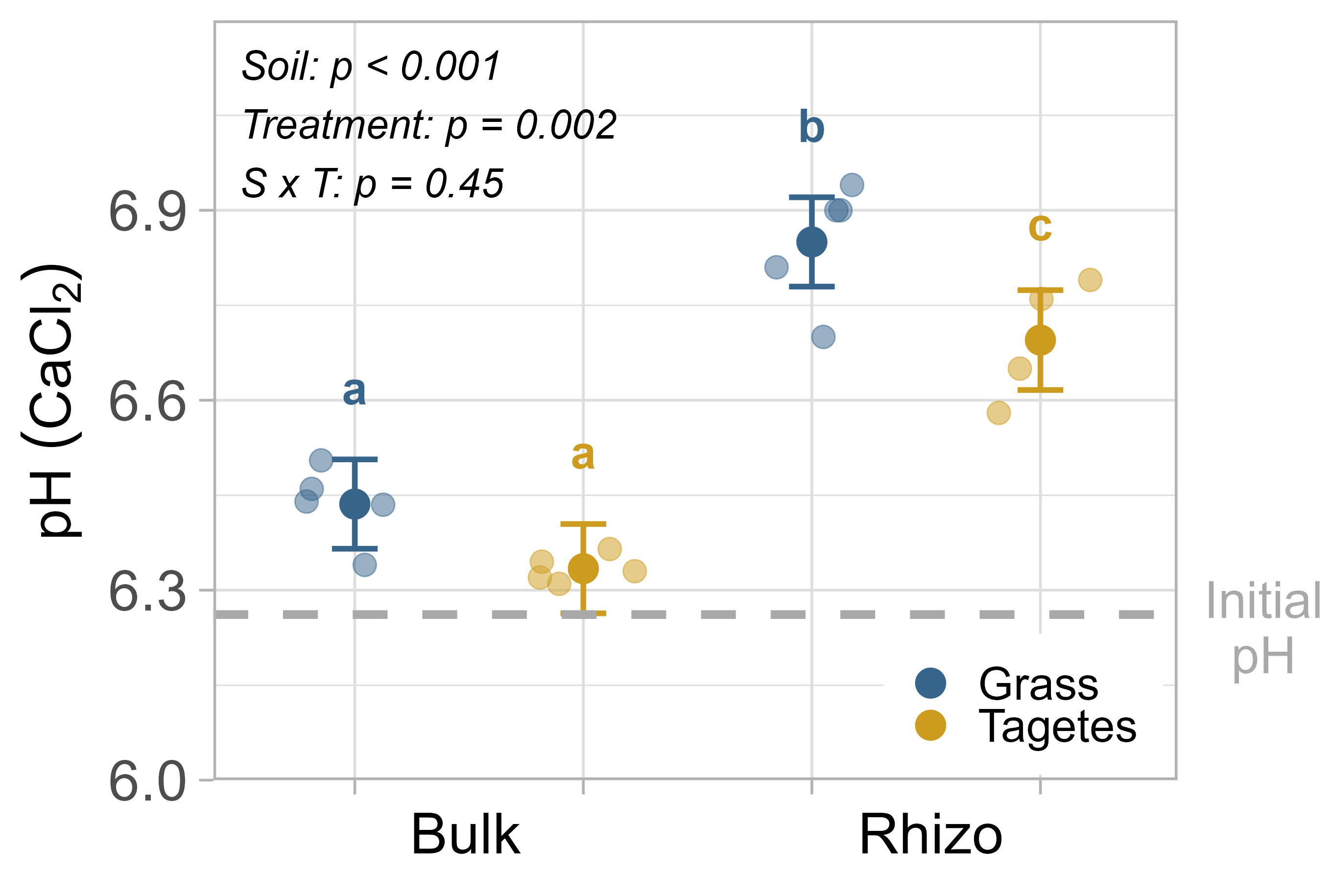
**

**S8 Fig.** **Jitter plots of soil pH measured in bulk and rhizosphere soil from columns planted with grass or Tagetes patula.** “Initial pH” indicates soil pH prior to planting. Jittered points show the values per column. Two-way ANOVA was performed to test for effects of culture (grass/*Tagetes*) and soil (rhizosphere/bulk). *P*-values from the ANOVA are shown in the upper left corner. Mean points represent predicted group means from the model; error bars indicate standard errors. Different letters illustrate significant differences (*p*<0.05).
