# Supplementary material for: Detecting apple replant disease in the field – deciphering reasons for local growth depression
Source: PLoS One. 2026 Apr 21;21(4):e0345851. doi: 10.1371/journal.pone.0345851 (PMC13098943; doi:10.1371/journal.pone.0345851)
Supplement: S1 Table — (DOCX) [file pone.0345851.s009.docx]

| **S1 Table.** **Biotest results as an indicator for ARD severity.** Mean reduction (%) in biomass (fresh matter) of M26 apple plantlets in untreated soil compared to the respective γ-irradiated soil (100 %). In relation to the entire plant, a reduction in biomass of up to 35 % was classified as “moderate”, from 36 to 45 % as “medium-severe”, and over 46 % as “severe” for this comparison. | | | | |
| --- | --- | --- | --- | --- |
| Site | Shoot | Root | Whole plant | ARD severity |
| HS | 47 | 49 | 48 | severe |
|  |  |  |  |  |
| BO | 37 | 35 | 36 | medium severe |
