# Supplementary material for: Detecting apple replant disease in the field – deciphering reasons for local growth depression
Source: PLoS One. 2026 Apr 21;21(4):e0345851. doi: 10.1371/journal.pone.0345851 (PMC13098943; doi:10.1371/journal.pone.0345851)
Supplement: S2 Table — (DOCX) [file pone.0345851.s010.docx]

| **S2 Table.** **Soil properties, ARD severity, and cultivation details for both study sites (SOC = soil organic carbon; N_t_ = total nitrogen).** | | | | | | | | | | | | | | |
| --- | --- | --- | --- | --- | --- | --- | --- | --- | --- | --- | --- | --- | --- | --- |
| Site | Clay (%) | Silt (%) | Sand (%) | Texture  (KA5, 2005) | SOC  (%) | | N_t_  (%) | ARD-severity | grass seeds | *Tagetes* seeds | Pre-culture sowing | Apple planting | Variety | rootstock |
| HS | 4.6 | 37.6 | 57.8 | medium silty sand | 1.36 | 0.12 | | severe | “Berliner Tiergarten Grassaat” | “Nemamix” | spring 2020 | March 2021 | “GS66” | M9 |
| BO | 7.4 | 28.3 | 64.3 | medium silty sand | 1.16 | 0.11 | | mediumsevere | common meadow-grass - *Poa pratensis* | “Nemamix” | spring 2019 | November 2019 | "Golden Delicious" (mutant Reinders) | M9 |
