# Supplementary material for: Detecting apple replant disease in the field – deciphering reasons for local growth depression
Source: PLoS One. 2026 Apr 21;21(4):e0345851. doi: 10.1371/journal.pone.0345851 (PMC13098943; doi:10.1371/journal.pone.0345851)
Supplement: S3 Table — (DOCX) [file pone.0345851.s011.docx]

| **S3 Table.** **Mean of single phytoalexins per tree group**. Values in µg/g dry matter (RI = retention index) | | | | | | | | | | | |
| --- | --- | --- | --- | --- | --- | --- | --- | --- | --- | --- | --- |
|  |  |  | HS | | | |  | BO | | | |
| phytoalexins | RI |  | control-better | control-  worse | *Tagetes*-better | *Tagetes*-worse |  | control-better | control-  worse | *Tagetes*-better | *Tagetes*-worse |
| 3,5-dihydroxybiphenyl | 2000 |  | 0 | 0 | 0 | 1.10 |  | 0 | 0 | 0 | 0 |
| aucuparin | 2090 |  | 2.30 | 31.29 | 21.23 | 52.15 |  | 18.28 | 10.52 | 25.23 | 55.09 |
| noraucuparin | 2121 |  | 5.35 | 15.16 | 10.63 | 16.24 |  | 2.19 | 3.14 | 15.43 | 17.72 |
| 2-hydroxy-4-methoxydibenzofuran | 2131 |  | 1.30 | 37.71 | 4.10 | 2.86 |  | 0 | 0 | 5.04 | 3.68 |
| 2'-hydroxyaucuparin | 2193 |  | 0 | 3.80 | 0 | 4.46 |  | 0 | 0 | 1.54 | 0 |
| methoxyeriobofuran isomer 2 | 2245 |  | 0 | 0 | 0 | 0 |  | 1.36 | 1.58 | 17.73 | 5.73 |
| noreriobofuran | 2259 |  | 0 | 12.95 | 3.00 | 3.38 |  | 1.88 | 3.72 | 12.35 | 2.45 |
| hydroxyeriobofuran isomer 1 | 2280 |  | 2.57 | 3.53 | 3.00 | 4.53 |  | 0 | 0 | 2.04 | 0 |
| noreriobofuran isomer 1 | 2285 |  | 0 | 0 | 0 | 8.36 |  | 0 | 0 | 2.83 | 0 |
| hydroxynoreriobofuran isomer 1 | 2286 |  | 1.66 | 2.85 | 2.73 | 6.96 |  | 0 | 1.33 | 1.45 | 1.89 |
| hydroxynoreriobofuran isomer 2 | 2314 |  | 0 | 0 | 0 | 0 |  | 0 | 0 | 0 | 1.16 |
| hydroxyeriobofuran isomer 2 | 2331 |  | 2.15 | 22.89 | 1.18 | 0.98 |  | 0 | 0.81 | 13.05 | 7.55 |
| eriobofuran isomer 2 | 2346 |  | 1.01 | 14.22 | 0 | 2.10 |  | 0 | 0 | 2.84 | 3.19 |
| hydroxynoreriobofuran isomer 4 | 2365 |  | 0 | 0 | 0 | 0 |  | 0 | 0 | 1.78 | 2.00 |
| hydroxyeriobofuran isomer 3 | 2461 |  | 0 | 2.59 | 0 | 0 |  | 0 | 0 | 8.66 | 0 |
| hydroxyeriobofuran isomer 5 | 2474 |  | 0 | 2.12 | 0 | 0 |  | 0.57 | 0 | 9.86 | 1.85 |
